# Long-Term Experience in the Molecular Genetic Diagnosis of Friedreich Ataxia in the Russian Federation

**DOI:** 10.3390/genes17101169

**Published:** 2026-09-23

**Authors:** Olga Ismagilova, Tagui Adyan, Nailya Galeeva, Viktoria Zabnenkova, Olga Shatokhina, Maria Kazaryan, Galina Rudenskaya, Elena Dadali, Elena Saifullina, Alexander Polyakov

**Affiliations:** 1 Research Centre for Medical Genetics, Moskvorechie Str., 1, 115522 Moscow, Russia; 2 Department of Medical Genetics and Fundamental Medicine, Bashkir State Medical University, Lenin Str., 3, 450008 Ufa, Russia

**Keywords:** Friedreich’s ataxia, GAA repeat expansion, molecular diagnosis

## Abstract

Background/Objectives: Friedreich’s ataxia in considered the most common form of autosomal recessive ataxia; however, it’s prevalence in the Russian Federation remains poorly studied. Methods: The study included 974 patient samples referred for analysis of the expanded GAA repeat in intron 1 of the *FXN* gene, as well as 319 healthy individuals participating in a biomaterial donation program for IVF, using PCR-AFLP and fragment analysis with labeled primers. Sanger sequencing of the *FXN* gene was additionally performed for six patients who exhibited a heterozygous expanded allele. Results: The diagnosis of FRDA was confirmed in 142 patients: 139 of them had homozygous GAA repeat expansions, the most common mutation type in this disorder, while in 3 cases a heterozygous expansion was associated with a pathogenic point mutation in the *FXN* gene on the second allele. For 28 probands, biallelic GAA expansions in *FXN* were subsequently confirmed in their affected relatives. Among the 319 healthy donors, only 2 carriers of a pathogenic expansion were identified. Conclusions: This paper presents the results of a long-term study on the molecular genetic causes of FRDA in Russian Federation, yielding an estimated frequency of 1:102,000 individuals for the disease associated with GAA repeat expansion.

## 1. Introduction

First described in the 19th century by Nikolaus Friedreich as a “degenerative atrophy of the posterior columns of the spinal cord,” Friedreich ataxia (FRDA) is now recognized as the most common form of autosomal recessive ataxia worldwide [1]. Epidemiological studies have reported a wide range of disease prevalence, varying from approximately 1:20,000 to 1:750,000 individuals depending on the population studied, with a global estimate of approximately 1:100,000 [2]. The carrier frequency of pathogenic *FXN* alleles is estimated to range from 1 in 60 to 1 in 120 individuals [3]. Data on the prevalence of FRDA in Russia remain limited. In particular, Kirilenko et al. (2004) estimated a prevalence of 1:200,000–1:330,000 based on epidemiological studies of neuromuscular disorders conducted in several regions of central and European Russia [4].

FRDA is caused by biallelic pathogenic variants in the *FXN* gene, located at chromosome locus 9q21.11, encodes frataxin, a mitochondrial protein that plays a crucial role in iron–sulfur cluster biogenesis, heme synthesis, and mitochondrial energy metabolism [5]. Although the full spectrum of frataxin functions requires further examination, the multisystem manifestations of FRDA are thought to result primarily from mitochondrial dysfunction, impaired oxidative phosphorylation, excessive production of free radicals, and increased susceptibility to oxidative stress, ultimately leading to progressive cellular degeneration [6].

The clinical features of FRDA include early disease onset and relatively rapid progression of neurological impairment compared with other hereditary ataxias, which are frequently accompanied by hypertrophic cardiomyopathy. The neurological manifestations predominantly include cerebellar and sensory ataxia, loss of deep tendon reflexes and proprioception, hypotonia, and progressive spasticity of the lower limbs [7]. Gait instability and frequent stumbling, typically developing during the first or second decade of life, are among the earliest clinically recognizable manifestations. However, diminished tendon reflexes and impaired proprioception generally precede the onset of overt ataxia [8]. The size of the GAA repeat expansion is strongly associated with both the age at disease onset and clinical severity [9].

The vast majority of FRDA cases (up to 96%) are caused by an expanded GAA trinucleotide repeat located within an Alu repetitive element in intron 1 of the *FXN* gene. Expansion of the repeat disrupts pre-mRNA processing, resulting in a marked reduction in mature *FXN* mRNA levels [10]. Experimental studies have demonstrated substantially decreased frataxin expression in lymphoblastoid cells from affected individuals, ranging from 4% to 29% of normal levels, usually correlating with the size of the GAA expansion [11]. In unaffected individuals, the *FXN* allele typically contains 8–33 GAA repeats, whereas pathogenic alleles harbor approximately 66 to more than 1000 repeats. Intermediate alleles containing 34–65 GAA repeats are rare and are generally considered non-pathogenic; however, owing to their instability, they carry an increased risk of expansion in subsequent generations [12]. Long uninterrupted GAA repeat tracts exhibit pronounced somatic instability, whereas the presence of repeat interruptions by other nucleotides reduces the likelihood of further expansion during intergenerational transmission [13].

In approximately 4–5% of patients with FRDA, the genotype consists of a GAA repeat expansion on one *FXN* allele and a pathogenic sequence variant on the other allele [14]. Individuals carrying compound heterozygous pathogenic variants of different types may present with phenotypes that differ from the classical form of the disease, with the clinical manifestations depending on the nature of both variants. Earlier disease onset and a more severe clinical course have been associated with null variants causing complete cessation of frataxin production, whereas missense variants appear to exert variable effects depending on their location within the protein, their impact on protein stability, and the functional domains affected [15].

Intragenic deletions in *FXN* have also been reported and can be detected using quantitative multiplex ligation-dependent probe amplification (MLPA). Recently, long-read sequencing has enabled the identification of pathogenic deletions involving the *FXN* promoter region and exon 1. These deletions remove both the GAA repeat region and the primer-binding sites used in conventional PCR-based assays, potentially causing a hemizygous expanded allele to be misinterpreted as an apparently homozygous GAA repeat expansion [16]. Similar to truncating sequence variants that terminate frataxin expression, *FXN* deletions occurring in trans with a GAA expansion have been associated with earlier disease onset and more rapid clinical progression [17].

For many years, pharmacological management of FRDA was limited primarily to symptomatic treatment. Current gene-based therapeutic strategies are aimed at restoring frataxin expression through a variety of approaches; however, significant challenges remain, including vector-related toxicity and efficient delivery of therapeutic constructs to target tissues [18]. Given the central role of mitochondrial dysfunction in FRDA pathogenesis, considerable efforts have also focused on therapies that mitigate its consequences. These include compounds that enhance cellular metabolism, antioxidants, immunomodulators, deacetylase inhibitors, iron chelators, etc. [19].

Synthetic oleanane triterpenoid derivatives have demonstrated broad cytoprotective effects in both in vitro and in vivo models [20]. One of the most promising compounds in this class is omaveloxolone (OMA), which activates the nuclear factor erythroid 2-related factor 2 (Nrf2) signaling pathway, a master regulator of the cellular antioxidant response [21]. By activating Nrf2, OMA improves mitochondrial function, restores redox homeostasis, and suppresses the activation of nuclear factor-κB (NF-κB), thereby reducing oxidative stress and neuroinflammation [22]. Importantly, OMA has recently become the first and only approved disease-modifying therapy for FRDA in patients aged 16 years and older. Following its approval by the U.S. Food and Drug Administration (FDA) in 2023 and the European Commission in 2024, it is now available in over 40 countries and is being integrated into clinical practice. In Russia, a bioequivalence study of a generic omaveloxolone (OMV01) has recently been initiated.

## 2. Materials and Methods

Between 2006 and 2025, DNA samples from 974 individuals referred for *FXN* GAA repeat expansion testing were analyzed at the DNA Diagnostics Laboratory of the Research Centre for Medical Genetics (RCMG). The study cohort included 974 unrelated probands and 98 relatives from 53 families. Most patients had been referred with suspected early-onset neurodegenerative disorders, predominantly FRDA, unspecified hereditary ataxia, or Charcot–Marie–Tooth disease. In addition, data from 319 individuals referred through in vitro fertilization (IVF) gamete donor screening programs was included. Information on age and sex was unavailable for this group; however, all individuals were presumed to be clinically unaffected. Among the primary study cohort, the age at referral ranged from several months to 80 years (median, 16.5 years). Males slightly outnumbered females among patients with available sex data (535 vs. 436; male-to-female ratio, approximately 1.2:1).

Written informed consent for participation in the study and anonymous publication of the results was obtained from all participants or their legal guardians, as well as from participants in donor program.

Genomic DNA was extracted from peripheral whole blood collected in EDTA tubes using the Wizard^®^ Genomic DNA Purification Kit (Promega, Madison, WI, USA) according to the manufacturer’s instructions.

Until 2014, analysis was performed using a qualitative multiplex PCR-AFLP assay with primers flanking the GAA repeat region in intron 1 of the *FXN* gene, together with primers targeting exon 1 of the *HBB* gene, which served as an internal PCR amplification control. This approach allowed determination of the presence of at least one allele with a normal number of GAA repeats. In the absence of a visible *FXN* amplification product, biallelic GAA repeat expansions were presumed. The sequences of the primers used are provided in Table 1.

PCR was performed in a final reaction volume of 23 μL containing 1x reaction buffer (67 mM Tris-HCl, 16.6 mM (NH_4_)_2_SO_4_, 0.01% Tween-20), 0.06 μM of each primer, 250 μM of each dNTP, 75 pM MgCl_2_, 2.5 U of Biotaq thermostable DNA polymerase (Biomaster, Novosibirsk, Russia), 20–30 μL of mineral oil, and 2 μL of genomic DNA. Amplification was carried out on a Tercik programmable thermocycler (DNA-Technology, Moscow, Russia) with an initial denaturation step at 95 °C for 5 min, followed by 30 cycles of 94 °C for 2 s, 62 °C for 2 s, and 72 °C for 2 s under precise temperature control, with a final extension at 72 °C for 5 min.

PCR products were analyzed by vertical electrophoresis in 7% polyacrylamide gels (acrylamide/bis-acrylamide ratio 29:1), followed by staining with ethidium bromide and visualization using a GelDoc documentation system (Bio-Rad Laboratories, Hercules, CA, USA) with UV illumination (312 nm). Electropherograms for unaffected probands showed two bands corresponding to *HBB* exon 1 and the *FXN* intron 1 fragment when the GAA repeat size was within the normal or near-normal range (up to 60 repeats). Alleles carrying GAA repeat expansions (>200 trinucleotide repeats) are not routinely amplified under these conditions; therefore, samples harboring biallelic GAA repeat expansions yielded only the control *HBB* amplification product.

At present, molecular diagnosis of Friedreich ataxia is focused on detecting GAA repeat expansions and is performed using a two-step testing algorithm [23]. Since 2014, a second-stage assay has been incorporated into the diagnostic workflow to identify heterozygous carriers of the expanded allele by fragment analysis using a fluorescently labeled primer. Using PCR-AFLP and fragment analysis, 475 patients have been tested for GAA repeat expansions since 2014; no reanalysis of archival samples previously studied only by PCR-AFLP was performed.

The assay included three primers, one of which is complementary to both a fluorescently labeled primer and a sequence of 11 consecutive GAA repeats. This design generates multiple amplification products of varying lengths, depending on the annealing position of the repeat primer within the expanded region, the length of the repeat tract in the patient’s DNA, and the limitations of the amplification method. The primer sequences are listed in Table 1.

PCR was performed in a final reaction volume of 23 μL containing 1× reaction buffer (67 mM Tris-HCl, 16.6 mM (NH_4_)_2_SO_4_, 0.01% Tween-20), 0.4 μM of each primer, 250 μM of each dNTP, 135 pM MgCl_2_, 40 μM betaine, 2.5 U of Biotaq thermostable DNA polymerase (Biomaster, Novosibirsk, Russia), 20–30 μL of mineral oil, and 2 μL of genomic DNA. Amplification was carried out on a Tercik programmable thermocycler (DNA-Technology, Moscow, Russia) with an initial denaturation at 95 °C for 12 min, followed by 50 cycles of 94 °C for 45 s, 62 °C for 45 s, and 72 °C for 60 s, with a final extension at 72 °C for 5 min.

PCR products were analyzed by fluorescent fragment analysis on an ABI Prism 3500 Genetic Analyzer (Applied Biosystems, Waltham, MA, USA). Samples without GAA repeat expansions produced either a single peak (homozygous state) or a cluster of peaks (heterozygous state with different repeat lengths on the two chromosomes), corresponding to alleles within the normal repeat size range. In contrast, expanded alleles generated a characteristic continuous ladder of progressively decreasing peaks extending beyond the normal fragment size range. Although this method does not allow precise determination of the number of expanded GAA repeats, it complements the first-stage assay by enabling detection of heterozygous carriers. Together, the two-stage testing strategy allows determination of the presence of normal and expanded alleles and assessment of their zygosity (Figure 1).

Primers for both stages of the diagnostic assay were designed at the DNA Diagnostics Laboratory of the RCMG based on GenBank reference sequences and synthesized by CJSC Evrogen and RPC Sintol (Moscow, Russia).

For five patients in whom a GAA repeat expansion was identified on one allele, the coding region of the *FXN* gene (exons 1–5) was additionally analyzed by Sanger sequencing to detect pathogenic sequence variants on the second allele.

## 3. Results and Discussion

Among the 974 individuals examined, 142 (14.6%) received a molecular genetic diagnosis of Friedreich ataxia. Of these, 139 (97.9%) carried biallelic GAA repeat expansions, the most common mutation type causing this disorder. In addition, 14 heterozygous carriers of a pathogenic expanded GAA allele were identified. Among the five heterozygous GAA expansion carriers who subsequently underwent *FXN* Sanger sequencing (exons 1–5), three patients (2.1%) were found to harbor a pathogenic point variant on the second allele. In all three unrelated patients, the previously reported pathogenic nonsense variant c.157dupC, p.(Arg53Profs*40) was identified [24].

For 53 probands, biological samples from family members (parents or siblings) were additionally available for carrier testing or confirmation of familial FRDA, comprising 98 individuals in total. FRDA was confirmed in 43 individuals (including the probands) from 28 families. Thirteen families had two or three affected members; in one, the pathogenic variant was inherited from an affected parent, whereas the remaining familial cases involved affected siblings (Table 2).

The low confirmation rate can most likely be explained by the characteristics of the sample, as the study included all patients referred for *FXN* expansion testing. Among the referral diagnoses, in addition to suspected FRDA, there were also suspected early-onset neurodegenerative disorders, hereditary ataxia, or Charcot–Marie–Tooth disease in some patients. Therefore, we assumed that most patients had a clinical presentation of a neurological disorder and that the referral was driven by the need for differential diagnosis, as FRDA is the most common cause of autosomal recessive ataxia. However, the lack of clinical information beyond the referral diagnosis does not allow the studied sample to be considered specifically focused on FRDA.

The detection rate was also affected by the limitations of the PCR-AFLP method without additional fragment analysis: in patients examined before 2014, it was impossible to detect at least one expanded allele, which would have prompted further search for other types of pathogenic variants in the *FXN* gene.

Regarding the low carrier frequency in families referred for diagnosis, a second-step analysis capable of detecting heterozygous carriage of a single expanded allele was performed in relatives from only 23 of the 28 families with a confirmed diagnosis; 5 families were examined in the early years of the diagnostic program using PCR-AFLP only, and heterozygous carriage of a single expanded allele could not be recognized in 11 relatives (8 parents, one maternal grandfather, and 2 siblings), particularly in the parents of probands with biallelic expansion (or hemizygosity for the expanded allele with a deletion of the GAA repeat region on the second allele). Although these parents are presumed to be carriers of the GAA repeat expansion, they are not included in Table 2.

The spectrum of variants identified, with a predominance of GAA repeat expansions, is consistent with worldwide data. The methods used in this study do not allow detection of a possible hemizygous GAA expansion, and analysis for large deletions in the *FXN* gene was not performed in this study; however, further examination of this issue may be helpful. Failure to detect proximal *FXN* deletions does not affect molecular confirmation of the diagnosis in patients carrying a typical expansion on the second allele. However, when determining carrier status, such deletions remain undetectable by conventional PCR- and fragment analysis-based methods. Although these variants are rare, overlooking them may have important implications for reproductive risk assessment in affected families [16]. Furthermore, expanded alleles are associated with markedly reduced, but not completely absent, frataxin production. Consequently, alleles carrying null variants, including proximal *FXN* deletions, may have a less favorable prognosis with respect to therapeutic strategies aimed at stabilizing *FXN* mRNA or targeted excision of the expanded region within intron 1.

Among the 319 individuals referred for carrier screening of *FXN* GAA repeat expansions, only two heterozygous carriers of a pathogenic expansion were identified. This corresponds to an estimated carrier frequency of approximately 1 in 160 individuals in the Russian population. According to the Hardy–Weinberg law, this carrier frequency predicts an incidence of Friedreich ataxia caused by *FXN* GAA repeat expansions of approximately 1 in 102,000 individuals, which is 2–3 times higher than previous estimates based on patient detection rate during the examination conducted in several Russian regions between 1998 and 2000 [4]. The true disease frequency may be even higher because some patients may be compound heterozygous, carrying a point mutation on one allele and a GAA expansion on the other. The implementation of improved diagnostic methods and their increased accessibility may also have influenced the final results.

One of the limitations of this study is that geographic origin data were unavailable, as well as clinical information for most patients referred from different regions of the Russian Federation. Therefore, we could not assess regional differences in FRDA prevalence, identify subregions with potentially high levels of inbreeding and consanguinity, or draw any conclusions about the geographic distribution of the disease. It is also likely that the primary cohort included a proportion of apparently healthy individuals who were referred either for carrier testing in the absence of a known family history of Friedreich ataxia or for preconception carrier screening without this indication being specified in the referral documentation. Consequently, the results should not be considered an exact representation of the current epidemiology of Friedreich ataxia in Russia.

The heterogeneity of the study cohort is largely explained by the fact that, for many years, the RCMG served as the only institution in the Russian Federation providing routine molecular diagnostics for Friedreich ataxia, receiving patient samples from various regions. Although no previous large-scale studies of Friedreich ataxia have been conducted in the Russian population, the findings of the present study demonstrate that the mutational spectrum is generally consistent with that reported in other populations [4].

Since the discovery of GAA repeat expansions in the *FXN* gene as the genetic cause of Friedreich ataxia in 1996, both diagnostic and therapeutic approaches have advanced considerably. Modern molecular genetic techniques not only enable accurate confirmation of the diagnosis but also play an important preventive role in reproductive planning for carriers of pathogenic alleles. The early disease onset, rapid progression, and severe disability associated with FRDA, together with the increasing pace of disease-modifying therapy development, underscore the importance of early diagnosis and timely initiation of treatment. The introduction of effective therapies has the potential to substantially improve long-term outcomes for patients with FRDA.

## 4. Conclusions

This study presents a retrospective analysis of 20 years of molecular genetic diagnostics for Friedreich ataxia in Russia. In patients with suspected FRDA, testing for GAA repeat expansions in the *FXN* gene remains the fastest and most effective diagnostic approach. Over the 20-year study period, 157 patients received a molecularly confirmed diagnosis of FRDA at the DNA Diagnostics Laboratory of the RCMG. The carrier frequency of *FXN* GAA repeat expansions in the Russian population was estimated to be 1 in 160 individuals, corresponding to an estimated disease prevalence of approximately 1 in 102,000 individuals.

The worldwide distribution of FRDA is notably heterogeneous. The disease is exceptionally rare in sub-Saharan Africa and South Asia, with only a single family reported in Mali [25,26,27]. In North and South America, reported prevalence estimates range from 1:50,000 in the United States to 1:500,000 in Canada and Brazil, while a calculated frequency of 1:1,200,000 has been reported in Cuba [28,29,30,31]. Vankan (2013), summarizing population studies, described a clear west-to-east prevalence gradient in Europe, with the highest prevalence in northern Spain (1:21,000) and the lowest in Finland (1:750,000) [3]. Data from Eastern Europe indicate frequencies of 1:250,000 in Poland and 1:375,000 in the Czech Republic [3]. Previous data for the Russian population indicated a prevalence of 1:330,000 [4]; however, the data obtained in this study show a frequency almost three times higher, than the previously reported estimate. It should be noted that our sample may not be fully representative, as the referral diagnosis of FRDA was not specified for all samples sent for FXN expansion testing. The contribution of genetic heterogeneity among hereditary ataxias and other neurodegenerative disorders presenting with ataxia cannot be excluded, and the true prevalence may be higher.

The multisystem nature of FRDA reflects the broad range of intracellular functions performed by frataxin, and effective treatment will therefore require a comprehensive therapeutic approach. The ongoing development of therapies targeting different pathogenic mechanisms is of great importance for patients and their families, as is improving access to molecular diagnostics and treatment. As disease-modifying therapies become available, the ability to slow disease progression and improve patients’ quality of life is becoming a reality. Accurate molecular confirmation of the diagnosis is therefore essential for appropriate therapeutic decision-making.

## Figures and Tables

**Figure 1:**
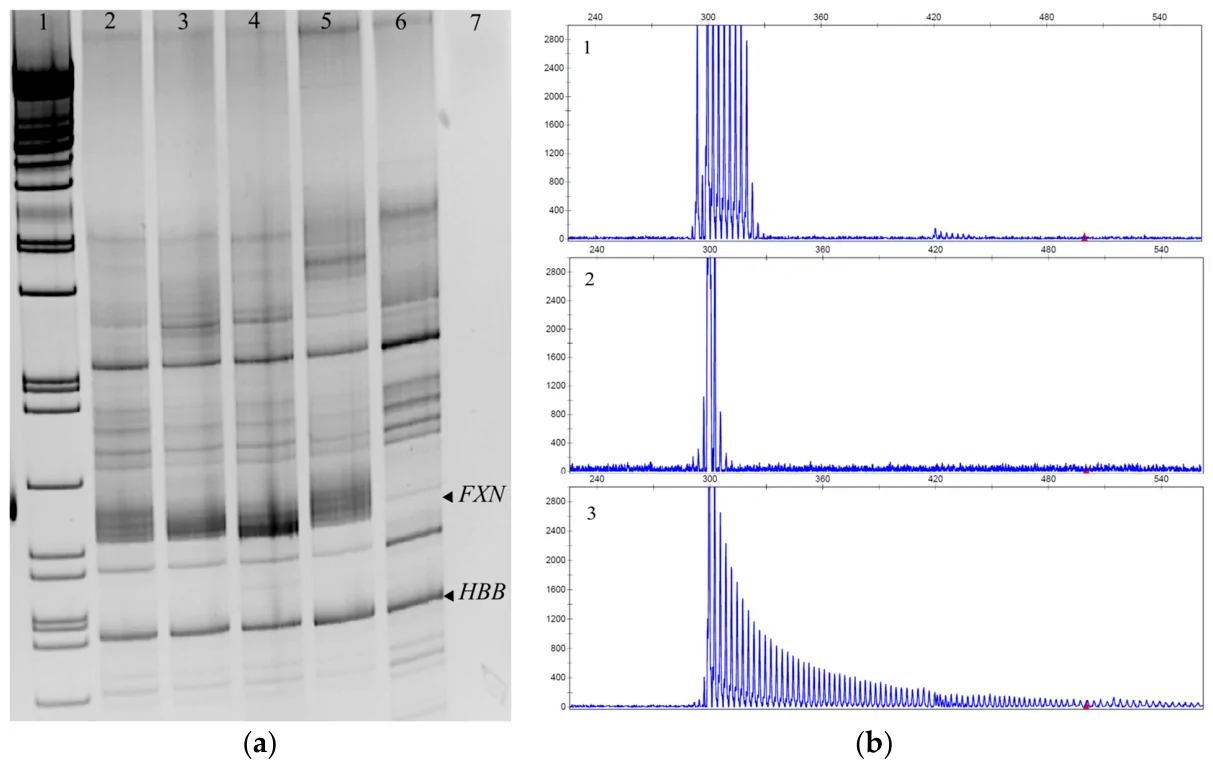
(**a**) Visualization of the first-stage assay (multiplex PCR-AFLP). Lane 1—molecular weight marker; lanes 2–5—samples with a detectable *FXN* allele of normal length; lane 6—absence of an amplified *FXN* fragment, indicating presumed biallelic GAA repeat expansion; lane 7—negative control. (**b**) Visualization of the second-stage assay (fragment analysis). 1 and 2—alleles within the normal GAA repeat size range; 3—the sample demonstrates an expanded GAA repeat on at least one allele.

**Table 1:** Primer sequences used for the reaction (5′ → 3′).

Stage 1	Anneal T °C
AFF: CTTTGGGATTGGTTGCCAGTGC	62
AFR: GACCATCATGGCCACACTTGCC	
SDHBB: FACTCCTGAGGAGAAGTCTGCC	
SDHBBR: AAGGTGCCCTTGAGGTTGTCC	
Stage 2	Anneal T °C
F-Fam: GTTCGTACGTGAATCGCGGTAC	65
AF FL: GTTCGTACGTGAATCGCGGTAC	
AAGAAGAAGAAGAAGAAGAAGAAGAAGAAGAAGAA	
AFR: GACCATCATGGCCACACTTGCC	

**Table 2:** Russian cohort analysis results.

	Number of Patients	Diagnosis Established	Genotype		
			Biallelic Expansion	Expansion + Point Mutation	Carriers
Referred patients	974	142	139	3	14
family members	53	15	15	-	22
IVF donors	319	-	-	-	2

## Data Availability

The original contributions presented in the study are included in the article; further inquiries can be directed to the corresponding author.

## References

[1] Friedreich N. (1863). Ueber degenerative Atrophie der spinalen Hinterstränge. Arch. Pathol. Anat. Physiol. Klin. Med..

[2] Buesch K., Zhang R. (2022). A systematic review of disease prevalence, health-related quality of life, and economic outcomes associated with Friedreich’s Ataxia. Curr. Med. Res. Opin..

[3] Vankan P. (2013). Prevalence Gradients of Friedreich’s Ataxia and R1b Haplotype in Europe Co-Localize, Suggesting a Common Palaeolithic Origin in the Franco-Cantabrian Ice Age Refuge. J. Neurochem..

[4] Kirilenko N.B., Fedotov V.P., Baryshnikova N.V., Dadali E.L., Poliakov A.V. (2004). Nozological spectrum of hereditary diseases of the nervous system in the cities of Volgograd and Volzhsky. Russ. J. Genet..

[5] Campuzano V., Montermini L., Moltò M.D., Pianese L., Cossée M., Cavalcanti F., Monros E., Rodius F., Duclos F., Monticelli A. (1996). Friedreich’s ataxia: Autosomal recessive disease caused by an intronic GAA triplet repeat expansion. Science.

[6] González-Cabo P., Palau F. (2013). Mitochondrial pathophysiology in Friedreich’s ataxia. J. Neurochem..

[7] Lynch D.R., Schadt K., Kichula E., McCormack S., Lin K.Y. (2021). Friedreich Ataxia: Multidisciplinary Clinical Care. J. Multidiscip. Healthc..

[8] Marty B., Naeije G., Bourguignon M., Wens V., Jousmäki V., Lynch D.R., Gaetz W., Goldman S., Hari R., Pandolfo M. (2019). Evidence for genetically determined degeneration of proprioceptive tracts in Friedreich ataxia. Neurology.

[9] Benkirane M., Marelli C., Choumert A., Goizet C., Patat O., Ewenczyk C., Anheim M., Mégarbané A., Larrieu L., Charlin C. (2025). Type and position of repeat interruptions as determinants of disease severity and expansion size in Friedreich ataxia. Genet. Med..

[10] Baralle M., Pastor T., Bussani E., Pagani F. (2008). Influence of Friedreich ataxia GAA noncoding repeat expansions on pre-mRNA processing. Am. J. Hum. Genet..

[11] Campuzano V., Montermini L., Lutz Y., Cova L., Hindelang C., Jiralerspong S., Trottier Y., Kish S.J., Faucheux B., Trouillas P. (1997). Frataxin is reduced in Friedreich ataxia patients and is associated with mitochondrial membranes. Hum. Mol. Genet..

[12] Sharma R., De Biase I., Gomez M., Delatycki M.B., Ashizawa T., Bidichandani S.I. (2004). Friedreich ataxia in carriers of unstable borderline GAA triplet-repeat alleles. Ann. Neurol..

[13] Montermini L., Kish S.J., Jiralerspong S., Lamarche J.B., Pandolfo M. (1997). Somatic mosaicism for friedreich’s ataxia GAA triplet repeat expansions in the central nervous system. Neurology.

[14] Galea C.A., Huq A., Lockhart P.J., Tai G., Corben L.A., Yiu E.M., Gurrin L.C., Lynch D.R., Gelbard S., Durr A. (2016). Compound heterozygous FXN mutations and clinical outcome in friedreich ataxia. Ann. Neurol..

[15] Shen M.M., Rummey C., Lynch D.R. (2024). Phenotypic variation of FXN compound heterozygotes in a Friedreich ataxia cohort. Ann. Clin. Transl. Neurol..

[16] Devore M.C., Lam C., Wiley G., Park C.C., Lynch D.R., Bidichandani S.I. (2026). Unrecognized high prevalence of expanded composite repeats in Friedreich ataxia. Hum. Mol. Genet..

[17] Hoffman-Zacharska D., Mazurczak T., Zajkowski T., Tataj R., Gorka-Skoczylas P., Polatynska K., Kępczyński Ł., Stasiołek M., Bal J. (2016). Friedreich ataxia is not only a GAA repeats expansion disorder: Implications for molecular testing and counselling. J. Appl. Genet..

[18] Sivakumar A., Cherqui S. (2022). Advantages and Limitations of Gene Therapy and Gene Editing for Friedreich’s Ataxia. Front. Genome Ed..

[19] Scott V., Delatycki M.B., Tai G., Corben L.A. (2024). New and Emerging Drug and Gene Therapies for Friedreich Ataxia. CNS Drugs.

[20] Jannus F., Sainz J., Reyes-Zurita F.J. (2024). Principal bioactive properties of oleanolic acid, its derivatives, and analogues. Molecules.

[21] Cordaro M., Neri G., Ansari S.A.M.K., Buccheri R., Scala A., Piperno A. (2025). Synthesis and Biological Profile of Omaveloxolone: The Cornerstone for Friedreich Ataxia Treatment. Int. J. Mol. Sci..

[22] Perlman S., Anheim M., Boesch S., Lewis J.H., Lynch D.R. (2025). Managing Aminotransferase Elevations in Patients with Friedreich Ataxia Treated with Omaveloxolone: A Review and Expert Opinion on Use Considerations. Neurol. Ther..

[23] Galeeva N.M., Mironovich O.L., Zabnenkova V.V., Shchagina O.A., Polyakov A.V. DNA Diagnosis of Friedreich’s Ataxia Using a New Medical Technology “Detection GAA Expansion in FXN Gene, Liability for Friedreich’s Ataxia”. https://www.medgen-journal.ru/jour/article/view/95/83.

[24] Cossee M., Dürr A., Schmitt M., Dahl N., Trouillas P., Kostrzewa M., Nivelon-Chevallier A., Gustavson K.H., Kohlschütter A., Müller U. (1999). Friedreich’s ataxia: Point mutations and clinical presentation of compound heterozygotes. Ann. Neurol..

[25] Cissé C.A.K., Cissé L., Bah H.O., Samassékou O., Simaga A., Tamega A., Diarra S., Diallo S.H., Coulibaly T., Diallo S. (2021). Friedreich ataxia in a family from Mali, West Africa. Clin. Case Rep..

[26] Labuda M., Labuda D., Miranda C., Poirier J., Soong B.W., Barucha N.E., Pandolfo M. (2000). Unique origin and specific ethnic distribution of the Friedreich ataxia GAA expansion. Neurology.

[27] Zeng J., Wang J., Zeng S., He M., Zeng X., Zhou Y., Liu Z., Jiang H., Tang B. (2015). Friedreich’s Ataxia (FRDA) is an extremely rare cause of autosomal recessive ataxia in Chinese Han population. J. Neurol. Sci..

[28] Keita M., McIntyre K., Rodden L.-N., Schadt K., Lynch D.R. (2022). Friedreich ataxia: Clinical features and new developments. Neurodegener. Dis. Manag..

[29] Salem I.H., Beaudin M., Stumpf M., Estiar M.A., Côté P.-O., Brunet F., Gamache P.-L., Rouleau G.A., Mourabit-Amari K., Gan-Or Z. (2021). Genetic and epidemiological study of adult ataxia and spastic paraplegia in Eastern Quebec. Can. J. Neurol. Sci..

[30] Fussiger H., Saraiva-Pereira M.L., Leistner-Segal S., Jardim L.B. (2019). Friedreich ataxia: Diagnostic yield and minimal frequency in South Brazil. Cerebellum.

[31] Velazquez-Perez L., Medrano-Montero J., Rodriguez-Labrada R., Canales-Ochoa N., Campins Ali J., Carrillo Rodes F.J., Rodriguez Grana T., Hernandez Oliver M.O., Aguilera Rodriguez R., Dominguez Barrios Y. (2020). Hereditary Ataxias in Cuba: A Nationwide Epidemiological and Clinical Study in 1001 Patients. Cerebellum.

